# Hexagonal Hollow‐Core Photonic Crystal Fiber for High‐Sensitivity Terahertz Glucose Detection

**DOI:** 10.1002/jbio.70332

**Published:** 2026-07-30

**Authors:** Mohammad Abdullah‐Al‐Shafi, Shuvo Sen, Mashiyat Mubassera, Md. Tanvir Hossain Hawlader

**Affiliations:** ^1^ Faculty of Science and Engineering Southern Cross University Gold Coast Australia; ^2^ Department of Information and Communication Technology (ICT) Mawlana Bhashani Science and Technology University Tangail Bangladesh; ^3^ QC Analyst II, Ortec Inc Piedmont South Carolina USA; ^4^ Department of Applied Chemistry and Chemical Engineering Gopalganj Science and Technology University Gopalganj Bangladesh

**Keywords:** diabetes disease, machine learning, photonic crystal fiber, sensor, THz

## Abstract

Diabetes mellitus is a rapidly escalating global health concern, often leading to severe complications such as neuropathy, vision impairment, vascular dysfunction, and renal failure. In this work, we present a newly designed hexagonal photonic crystal fiber (HPhCF) sensor for the noninvasive detection of urine glucose, offering a compact, fabrication‐friendly design. The proposed HPhCF demonstrates strong RS performance across a range of analyte concentrations. Specifically, RS values of 96.90%, 96.86%, 96.40%, 96.35%, 95.88%, and 95.78% are obtained for concentrations of 0–15 mg·dL^−1^, 0.625 g·dL^−1^, 1.25 g·dL^−1^, 2.5 g·dL^−1^, 5 g·dL^−1^, and 10 g·dL^−1^, respectively. These results indicate a gradual decline in sensitivity with increasing analyte concentration, while overall maintaining consistently high performance. Because of its simple structural configuration and compatibility with established photonic fabrication methods, the proposed sensor demonstrates strong potential as a low‐cost and highly sensitive diagnostic tool for early‐stage diabetes screening and ongoing glucose monitoring in biomedical settings.

## Graphical abstract

A novel hexagonal hollow‐core photonic crystal fiber (HPhCF) operating at 2.2 THz is proposed for rapid, non‐invasive detection of urinary glucose. The Zeonex‐based hollow‐core architecture enhances light‐analyte interaction, enabling 96.90% relative sensitivity while maintaining ultra‐low confinement loss and low effective material loss. Finite element simulations demonstrate stable optical performance over clinically relevant glucose concentrations, highlighting the potential of the proposed sensor for next‐generation terahertz biomedical diagnostics.

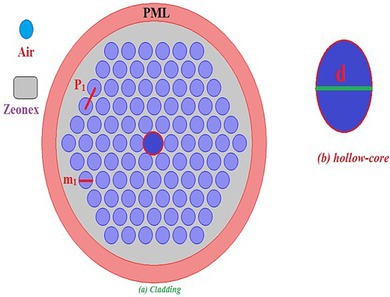

## 1 Introduction

Diabetes mellitus (DM) is a long‐term metabolic condition characterized by persistent disturbances in glucose regulation arising from insufficient insulin secretion or impaired insulin utilization, resulting in persistently elevated glucose levels in the bloodstream. The onset of diabetes can be attributed to a combination of genetic predisposition, sedentary lifestyle patterns, poor dietary habits, and other physiological or pathological conditions that disrupt insulin regulation. The global prevalence of diabetes has now surpassed 500 million cases, a figure forecasted to escalate by approximately 25% over the next decade [1]. The elevation of blood glucose levels primarily stems from reduced insulin synthesis or activity. Glucose appears in the urine when blood glucose levels rise above the renal threshold (8.8–10 mmol/L) or when the renal tubules are unable to adequately reabsorb filtered glucose, resulting in marked urinary glucose excretion [2]. Prolonged hyperglycemia further impairs immune function and damages the vasculature, thereby reducing the body's ability to resist infections and increasing vulnerability to diseases such as COVID‐19 [3]. Monitoring glucose concentration in urine, therefore, provides an effective means of assessing renal function decline beyond traditional blood glucose measurements [4]. Although both blood and urine glucose analyses are viable for diagnosing diabetes, blood‐based methods are often more expensive and demand greater technical expertise [5]. In contrast, urine glucose testing offers a cost‐effective, noninvasive, and user‐friendly alternative that individuals can perform at home to routinely monitor disease progression [6]. This approach is particularly advantageous in low‐resource settings, as it alleviates economic and healthcare burdens while facilitating early detection and management. Hence, developing a reliable and quantitative method for urine glucose analysis holds both scientific and clinical importance.

Photonic crystal fibers (PhCF) utilize a micro‐structured array of longitudinal air holes to achieve precise control over optical confinement and dispersion [7, 8]. This structural engineering makes them exceptionally well‐suited for guiding terahertz (THz) waves. Occupying the spectrum between microwaves and infrared radiation, the 0.1 to 10 THz band possesses unique propagation characteristics that have driven significant research in high‐speed communications, security, and spectroscopy [9, 10, 11, 12, 13, 14, 15, 16]. Consequently, PhCFs have emerged as a highly effective platform for the optical detection of chemical and biological analytes within this frequency range [17, 18]. To enhance their performance, researchers have employed a variety of low‐loss substrate materials such as Teflon, TOPAS, and ZEONEX, which effectively minimize optical attenuation and improve transmission efficiency [13, 19]. These materials have demonstrated promising potential in achieving key performance indicators, including reduced confinement loss (CL), elevated relative sensitivity (RS), and enlarged effective area (EA) [7, 8, 9, 10, 11, 12, 13, 14, 15, 16].

Consequently, there exists a compelling need to engineer a next‐generation PhCFs capable of simultaneously delivering low propagation losses and enhanced sensitivity within the THz regime for a broad spectrum of sensing applications [20]. DM, a rapidly escalating global health concern, currently affects approximately 415 million individuals worldwide and remains a leading cause of mortality among adults aged 20–60 years [21]. DM imposes a rapidly growing burden on global health, currently affecting an estimated 415 million individuals. Moreover, it remains a primary driver of mortality among adults aged 20–60 years [21]. The disease poses severe complications, including visual impairment, vascular dysfunction, renal failure, neurological disorders, and increased mortality risk [3, 22]. Moreover, it contributes significantly to limb amputations and is closely associated with cardiac and renal pathologies that elevate global death rates [23]. Diverse optical and electrochemical modalities have been investigated for glucose monitoring, including Fourier Transform Infrared, Raman, and Near‐Infrared spectroscopy, alongside reverse iontophoresis [24, 25, 26]. However, these approaches are often hindered by issues such as complexity, low accuracy, and lengthy analysis time [27].

To overcome these limitations, this research suggests an innovative hexagonal photonic crystal fiber (HPhCF) designed for precise, rapid, and highly sensitive glucose detection in the THz domain. The sensor is modeled and analyzed at 2.2 THz using the Finite Element Method (FEM), as executed in COMSOL Multiphysics. Key performance parameters, including CL, EA, and RS, are systematically evaluated and benchmarked against existing PhCFs designs. At the operating frequency of 2.2 THz, the proposed HPhCF exhibits RS values of 96.90%, 96.86%, 96.40%, 96.35%, 95.88%, and 95.78% at analyte concentrations of 0–15 mg·dL^−1^, 0.625 g·dL^−1^, 1.25 g·dL^−1^, 2.5 g·dL^−1^, 5 g·dL^−1^, and 10 g·dL^−1^, separately. The results demonstrate the superior efficiency and diagnostic potential of the proposed HPhCF, highlighting its promise as a viable platform for advanced biochemical sensing in future applications.

## 2 Methodology of the Designed Sensor

This study introduces the design of a novel, homogeneous, and structurally simple HPhCF. The proposed sensor was formulated and critically examined with Finite Element Analysis (FEA) through COMSOL Multiphysics to ensure precise evaluation and optimization of its optical characteristics. The unique structure of the sensor incorporates a hexagonal form, forming a distinct cladding region, while a hollow core is positioned at the center of the fiber. This geometric configuration offers enhanced light confinement and improved sensing stability. Zeonex, a high‐performance polymer, is deployed as the substrate material for the sensor's microstructure. This polymer is chosen for its excellent optical clarity, strong mechanical durability, minimal optical absorption, and inherent biocompatibility, all of which render it well‐suited for use in biochemical and environmental sensing applications [28]. Moreover, Zeonex exhibits excellent chemical resistance and mechanical robustness, ensuring long‐term operational stability and resistance to environmental degradation [13, 16]. However, in comparison with conventional materials such as silica or polymethyl methacrylate, Zeonex presents certain fabrication challenges and limited compositional tunability [29]. In this study, the RI of Zeonex is assumed to be 1.53. The designed cladding region contains a total of 90 composite air holes, which contribute to the fiber's low CL and enhanced light‐matter interaction. Air, with a refractive index (RI) = 1.00, fills the cladding holes, while glucose samples with varying refractive indices (RI = 1.335–1.347) are introduced into the core region to simulate biochemical sensing conditions. The interaction between guided modes and glucose‐filled regions enables precise RI detection, highlighting the potential of the HPhCF for glucose monitoring and other biosensing applications. To ensure consistent accuracy and sensitivity, periodic calibration and performance monitoring are essential, particularly when addressing factors such as material aging and potential contamination [30]. Overall, the incorporation of Zeonex as the structural medium, coupled with the optimized HPhCF design, demonstrates a robust and efficient optical platform suitable for reliable, long‐term sensing applications.

**FIGURE 1:**
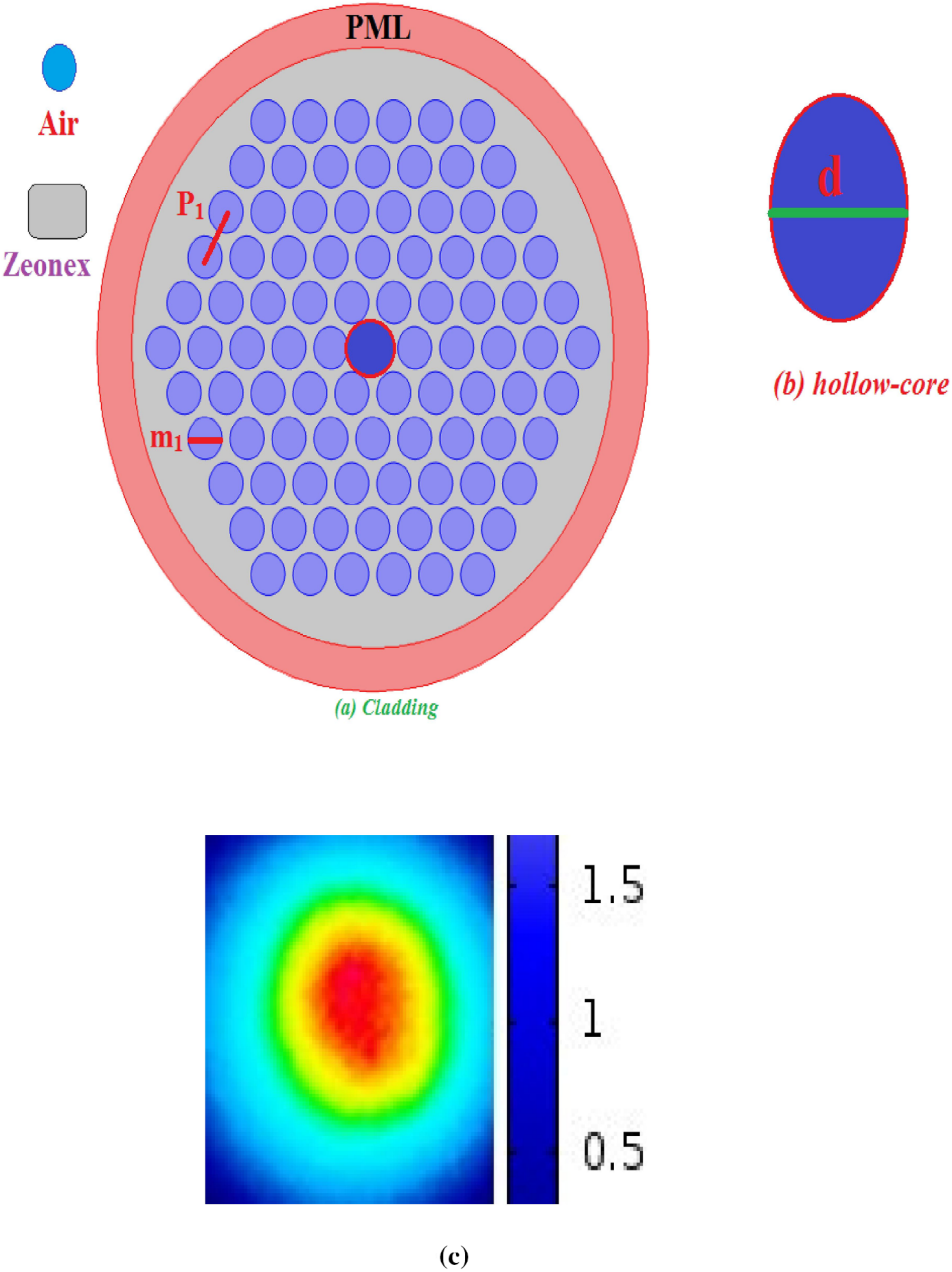
Proposed sensor geometry and energy distribution at 2.2 THz: (a) cladding structure, (b) hollow‐core region, (c) spectral energy allocation.

The two‐dimensional cross‐sectional geometry of the proposed HPhCF sensor is schematically illustrated in Figure 1. The structure features a central hollow core, denoted by *d* ^a^, with a width of 86 μm. Surrounding the core, the cladding is configured with five concentric rings of air holes, where d_1_ and A_1_ define the air hole diameter and the lattice pitch, respectively. To ensure the fidelity of the numerical modeling, a Perfectly Matched Layer (PML) boundary condition with a thickness equal to 10% of the fiber's maximum transverse dimension was employed to effectively suppress spurious reflections. The optimized geometric parameters, detailed in Table 1, were determined as follows: a hollow core diameter (*d*) of 86 μm, a cladding air hole diameter (*d*_1_) of 365 μm, and a uniform cladding pitch (A_1_ through A_5_) of 455 μm.

**TABLE 1:** Evaluation of critical parameters and performance metrics for the HPhCF configuration.

Specifications	Geometric parameters (μm)	Design attributes	Performance analysis
Cladding pitch (*A*_1_)	455	Sensitivity (0–15 mg·dL^−1^)	96.90%
Cladding distance (*d*_1_)	365	Operative region (0–15 mg·dL^−1^)	7.10 × 10^−8^ m^2^
Hollow core size (*d*)	86	CL (0–15 mg·dL^−1^)	6.15 × 10^−8^ dB/m
PML_1_	2460	—	—
PML_2_	2706	—	—

Figure 1c illustrates the optical field distribution of the guided mode across specific analyte RIs (1.335, 1.336, 1.337, 1.338, 1.341, and 1.347). These values correspond to urine glucose concentrations ranging from 0–15 mg·dL^−1^ to 10 g·dL^−1^, as follows: detailed in Table 2. The visualization confirms that the electromagnetic field remains tightly confined within the core throughout these variations, validating the proposed HPhCF's robust light‐guiding performance and high sensing efficiency.

**TABLE 2:** The RIs for various glucose concentrations in urine samples [30].

Glucose level (*C*)	RI
0–15 mg/dL	1.335
0.625 g/dL	1.336
1.25 g/dL	1.337
2.5 g/dL	1.338
5 g/dL	1.341
10 g/dL	1.347

**FIGURE 2:**
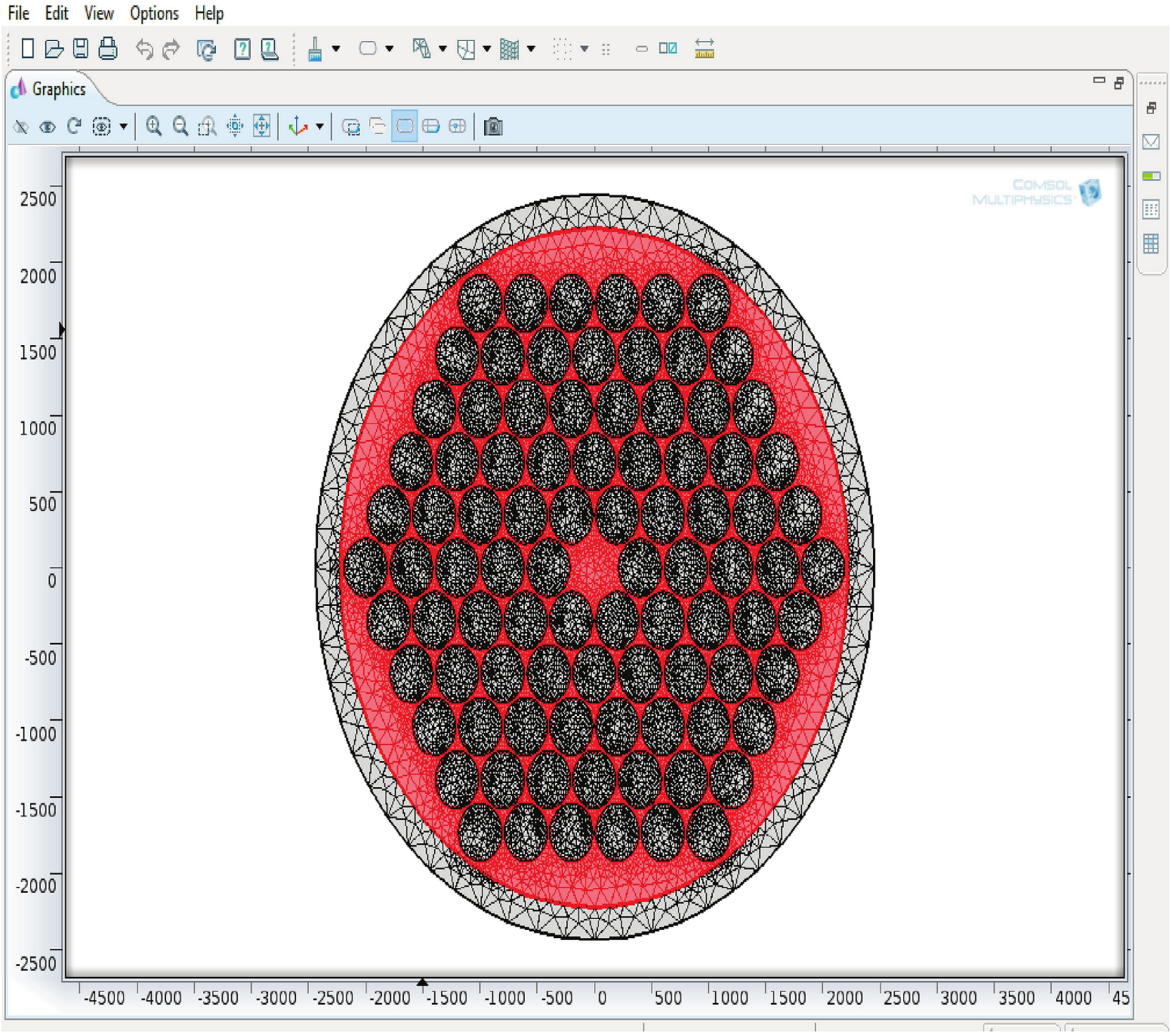
High‐fidelity mesh‐based modeling of implicit hollow‐core configurations.

Figure 2 presents the meshing profile of the simulated structure. The optimized model exhibits excellent meshing quality, comprising 18 225 domain elements and 1845 boundary elements, ensuring high numerical accuracy and stable convergence during simulation.

## 3 System Configuration

The framework for analyzing the functionality of the proposed HPhCF was established using an FEA implemented in COMSOL Multiphysics. The sensor model was designed to evaluate its optical response to various glucose concentrations in urine within the THz frequency domain, particularly in the 1–3 THz range. A two‐dimensional cross‐sectional structure of the HPhCF was developed to mimic realistic propagation behavior. The core region comprised a single hollow filled with the analyte (urine sample with varying glucose levels), surrounded by hexagonal air holes forming the cladding.

**FIGURE 3:**
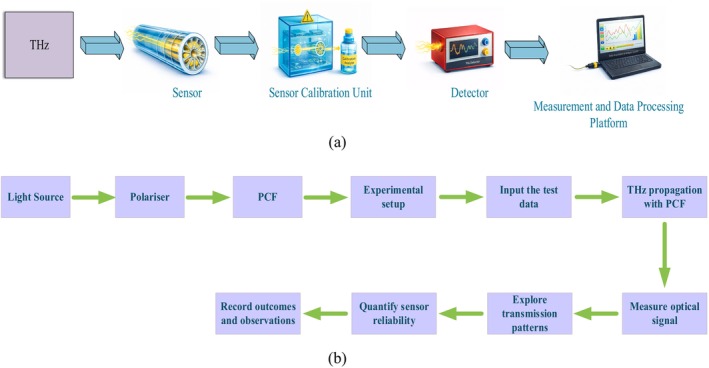
Experimental configuration for the designed PhCF (a) external layout (b) internal layout.

Figure 3 delineates the experimental framework of the optimized hollow‐core hexagonal PhCF sensor, specifically engineered for high‐precision glucose detection. Figure (a) presents the external view of the design, and (b) presents the internal layouts. The experimental arrangement comprised a broadband laser source, single‐mode fiber (SMF), and an optical spectrum analyzer (OSA). Light launched from the source propagated along the SMF and was subsequently coupled into the hollow‐core PhCF. Crucially, the PhCF incorporates a selectively etched sensing region to maximize the communication between the guided light and the glucose biomarker. Post‐transmission, the modulated light is captured by the OSA, with the resulting spectral data transferred to a processing unit for in‐depth interpretation. The upper inset of Figure 3 reveals the fiber's microstructural architecture, featuring a periodic hexagonal lattice of air holes that encircles the central hollow core. Key geometric parameters, specifically the diameter of air holes (d) and pitch (Λ), are clearly demarcated to define the guiding properties. To ensure the integrity of the numerical analysis, a PML bounds the structure, effectively mitigating parasitic reflections during simulation. This arrangement offers a comprehensive perspective on the interplay between the photonic path and the configurational properties essential for high‐accuracy sensing.

During each simulation, the electric and magnetic field distributions were extracted to compute light confinement within the core. These field data were then used to determine the propagation characteristics and optical performance metrics based on standard electromagnetic relations. We determined the effective material loss (EML) using the Zeonex absorption coefficient (0.2 cm^−1^), while CL was derived from the imaginary component of the RI. RS was quantified by analyzing the power fraction ratio, specifically comparing the guided field within the biomarker region to the total optical power transmitted across the fiber. This comprehensive computational framework establishes a robust method for evaluating the HPhCF's sensing performance in well‐regulated THz conditions. The combination of COMSOL‐based FEA modeling and optimized geometric design enables accurate prediction of the sensor's response to RI variations associated with glucose concentrations, offering a reliable pathway toward high‐performance, noninvasive glucose monitoring.

In earlier developments, the fabrication of PhCF primarily relied on the conventional stack‐and‐draw technique [31]. However, this approach has proven inadequate for realizing PhCF featuring asymmetric or geometrically complex air hole arrangements. To overcome these limitations, several advanced fabrication methodologies, including sol–gel synthesis, mechanical drilling, extrusion‐based fabrication, and additive manufacturing techniques, have been explored [32]. These contemporary techniques exhibit strong potential for constructing intricate PhCF architectures. Although the sol–gel technique is highly effective for creating circular pores in PhCFs, extrusion‐based fabrication and additive manufacturing techniques are preferred for fabricating non‐circular geometries, such as rectangular, square, or elliptical holes [33]. The HPhCF proposed herein is characterized by a hollow core encircled by a cladding of hexagonal air holes. Consequently, extrusion‐based fabrication and additive manufacturing techniques are identified as the most viable routes for realizing the proposed design. Notably, the Max Planck Institute has recently demonstrated the successful fabrication of asymmetric PhCF with diverse geometrical profiles, including rectangular and elliptical structures [34]. These achievements provide compelling experimental evidence supporting the fabrication feasibility of the proposed HPhCF architecture, affirming that such a hexagonal‐hole configuration can be reliably produced using current state‐of‐the‐art manufacturing techniques. Figure 3a,b illustrates the system setup for the proposed sensor.

## 4 Result Evaluation

A range of analytes is individually introduced into the core region of the proposed HPhCF to evaluate its sensing capability. Subsequently, the propagation of electromagnetic waves through the core gap of the designed HPhCF enables the assessment of several key performance parameters under different sensing media conditions. To investigate the effectiveness of the proposed HPhCF as an optical sensor, multiple performance metrics, including EA, numerical aperture (NA), EML, CL, P, and RS, are analyzed across the THz spectral region spanning 1–3 THz. This analysis focuses on detecting varying glucose concentrations in urine samples. The analysis was performed for urinary glucose concentrations of 0–15 mg/dL, 0.625 g/dL, 1.25 g/dL, 2.5 g/dL, 5 g/dL, and 10 g/dL.

**FIGURE 4:**
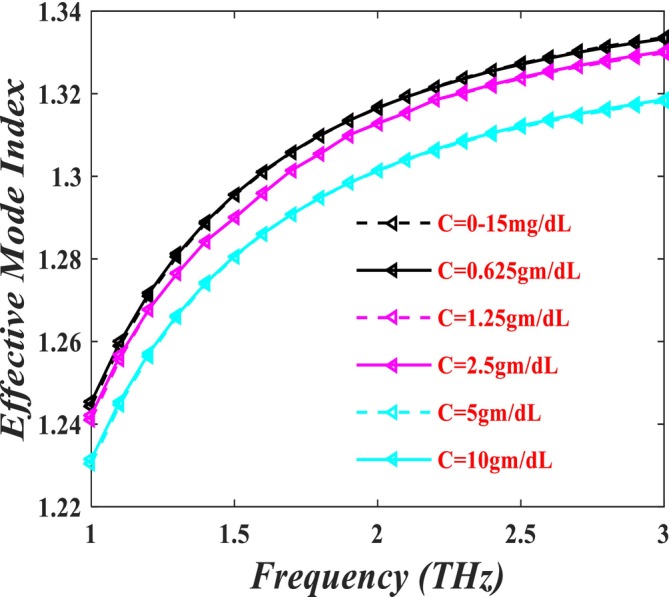
Frequency‐dependent characterization of the effective mode index.

The performance of the sensor is fundamentally governed by the effective RI (*n*_e_ff) of the guided mode. Figure 4 shows how *n*_e_ff varies across different sensing media at a fixed functional frequency of 2.2 THz, revealing a distinct dependence on glucose concentration. At this frequency, we calculated the *n*_e_ff assessments for urine samples containing glucose at concentrations of 0–15 mg/dL, 0.625 g/dL, 1.25 g/dL, 2.5 g/dL, 5 g/dL, and 10 g/dL, which are 1.3235, 1.3245, 1.3255, 1.3265, 1.3295, and 1.3354, respectively. Notably, these values remain identical, indicating polarization‐independent behavior of the sensor at the functional frequency. This consistent response highlights the capability of the designed HPhCF to accurately differentiate glucose concentrations in urine samples within the investigated THz spectrum.

To evaluate the operational efficiency of the proposed HPhCF, several key optical parameters must be determined. These parameters are assessed individually for each sensing medium. When the core gap is infiltrated with the sensing substance, an optical wave is launched and guided through the core region. Although the primary propagation occurs within the core, a portion of the electromagnetic field inevitably extends beyond it. The specific zone responsible for light confinement and interaction with the sensing medium is referred to as the EA. This parameter characterizes the spatial distribution of the optical field and operates as a determinant factor of light confinement within the fiber. Relation (1) defines the EA, where *E* represents the transverse component of the electric field.

(1) $$\mathrm{EA}=\frac{{\left(\int |E^{2}|\text{dxdy}\right)}^{2}}{\left(\int |E^{4}|\text{dxdy}\right)}\;m^{2}$$

In the formulation, [∫  $|E^{2}|\text{dxdy})$]^2^ reflects the joint influence of the spatial power distribution, while [∫  $|E^{4}|\text{dxdy})$] denotes the total accumulated field intensity across the entire domain.

**FIGURE 5:**
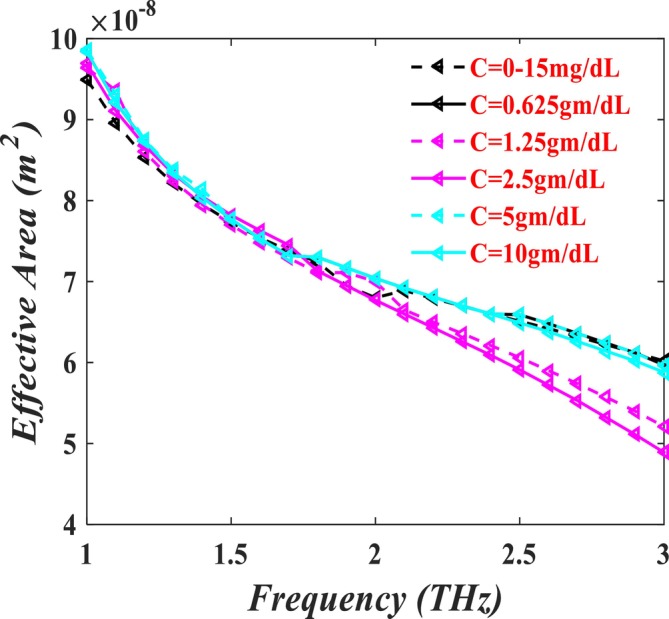
EA exhibits across the frequency spectrum.

Figure 5 presents the EA of the five analytes for the frequency spectrum. Lower EA values are desirable because the effective area determines how strongly the optical intensity field is constrained within the sensing region of the HPhCF structure. A smaller EA indicates stronger field confinement inside the core, which enhances light‐analyte interaction and improves sensing capability. At a functioning frequency of 2.2 THz, the EA values are 7.10 × 10^−8^ m^2^, 7.15 × 10^−8^ m^2^, 6.95 × 10^−8^ m^2^, 6.97 × 10^−8^ m^2^, 7.12 × 10^−8^ m^2^, and 7.16 × 10^−8^ m^2^ for biomarker content of 0–15 mg dL^−1^, 0.625 g dL^−1^, 1.25 g dL^−1^, 2.5 g dL^−1^, 5 g dL^−1^, and 10 g dL^−1^, correspondingly.

The range of incident wave angles that can be effectively coupled into and guided through an HPhCF is characterized by its NA. This parameter represents the light‐collecting capability of fiber and is expressed as a dimensionless quantity. NA is influenced by the EA, the functioning frequency (*f*), and the velocity of light (*c*). In sensing applications, a larger NA is generally suitable because it enhances the ability to capture incoming optical signals. Such an increase in NA can be achieved by reducing the EA of the fiber. The NA is calculated as follows: in Equation (2):

(2) $$\mathrm{NA}=\frac{1}{\sqrt{1+\frac{{\mathrm{\pi A}}_{\mathrm{eff}}f^{2}}{c^{2}}}}\approx \frac{1}{\sqrt{1+\frac{{\mathrm{\pi A}}_{\mathrm{eff}}}{\lambda^{2}}}}$$

**FIGURE 6:**
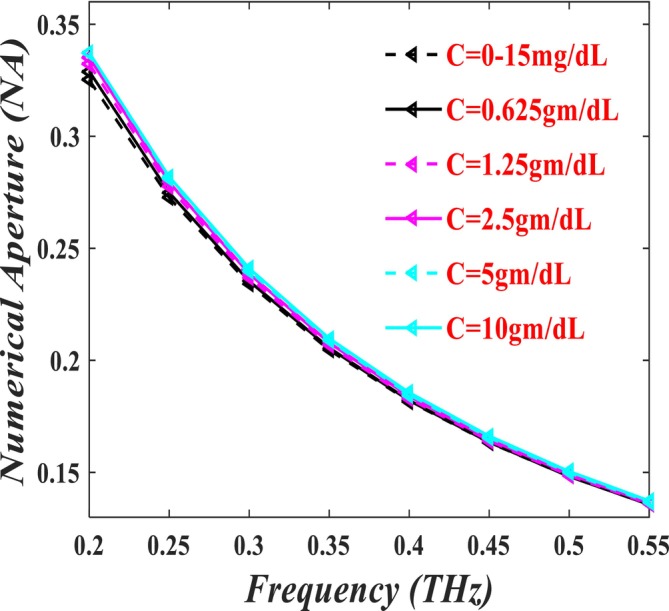
NA with THz frequency.

Figure 6 illustrates the deviation in NA regarding the operating frequency for the selected analytes. The results show a gradual increase in NA as the frequency increases. This trend is consistent with the theoretical relationship between NA and EA, where a higher NA is typically associated with stronger optical confinement and consequently a smaller effective area. At 0.35 THz, the calculated NA values for analyte content of 0–15 mg·dL^−1^, 0.625 g·dL^−1^, 1.25 g·dL^−1^, 2.5 g·dL^−1^, 5 g·dL^−1^, and 10 g·dL^−1^ are 0.23112, 0.24312, 0.25112, 0.25231, 0.26112, and 0.25711, respectively. These outcomes indicate that the structure keeps balanced optical characteristics across different analyte concentrations while demonstrating improved confinement behavior at higher frequencies.

The attenuation of optical power caused by the absorption characteristics of the cladding material in the HPhCF is referred to as EML. In the proposed refractive photonic crystal fiber sensor (HPhCF), Zeonex serves as the background substrate and is primarily responsible for this absorption‐induced loss. Consequently, reducing the proportion of background material within the fiber structure can effectively minimize the EML. This parameter can be measured with the formulation (3).

(3) $$\mathrm{EML}=\sqrt{\frac{{Ɛ}_{0}}{\mu_{0}}}\;\left(\frac{\int_{\mathrm{mat}}n_{\mathrm{mat}}{\left|E\right|}^{2}\;\alpha_{\mathrm{mat}}\mathrm{dA}}{.5*\left|\int_{\mathrm{all}}\left(E*\mathrm{H.z}\right)\mathrm{dA}\right|}\right)$$

In this context, $\varepsilon_{0}$ and $\mu_{0}$ refer to the relative permittivity and relative permeability of air, respectively. The parameters $\alpha_{\text{material}}$ and $\eta_{\text{material}}$ represent the bulk absorption coefficient (cm^−1^) and the RI of the cladding material, Zeonex. Furthermore, the Poynting vector, which describes the directional flow of electromagnetic energy, is denoted by z.

**FIGURE 7:**
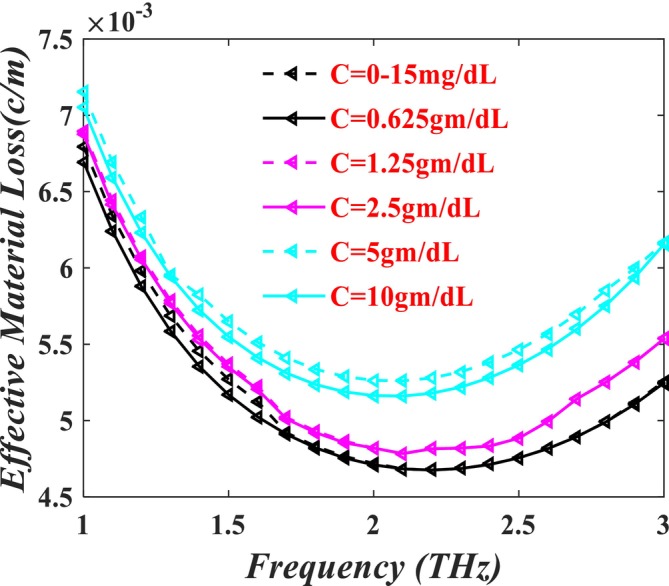
EML with THz frequency.

Figure 7 clarifies the EML of the designed HPhCF, respectively. At an optical frequency of 2.2 THz, the EML assessments gradually decrease with increasing concentration of the sensing medium. Specifically, the HPhCF exhibits EML values of approximately 0.00632, 0.00638, 0.00711, 0.00719, 0.00833, and 0.00838 cm^−1^ for analyte content of 0–15 mg·dL^−1^, 0.625 g·dL^−1^, 1.25 g·dL^−1^, 2.5 g·dL^−1^, 5 g·dL^−1^, and 10 g·dL^−1^, correspondingly.

As light propagates through an optical fiber, a segment of the optical power escapes from the core due to imperfect electromagnetic field confinement, leading to CL. In PhCF sensors, CL is a key parameter that directly affects sensing efficiency and accuracy. Losses attributed to geometric imperfections or coupling with the external medium can significantly impair the sensitivity of the device. Therefore, reliable performance evaluation requires careful assessment of CL. Additional losses may also occur due to system‐level leakage caused by design limitations or misalignment of structural openings. In PhCF structures, air‐filled microholes act as non‐conductive regions that help guide light while limiting undesired interactions. We quantified CL by modifying the fiber's geometric and material properties, utilizing the real and imaginary elements of the electromagnetic fields in conjunction with the EML, as formulated [35].

(4) LcdBm=8.686K0Imneff

(5) $$K_{0}=2\pi \left(\frac{f}{c}\right)$$

Equation (5) characterizes the guided wave by its frequency f and propagation velocity $c=3\times 10^{8}\;{\mathrm{ms}}^{-1}$. Here, $i_{m}$ in Equation (4) denotes the imaginary element of the wave representation, whereas $K_{0}$ in Equation (5) corresponds to the parameter governing intrinsic wave generation.

**FIGURE 8:**
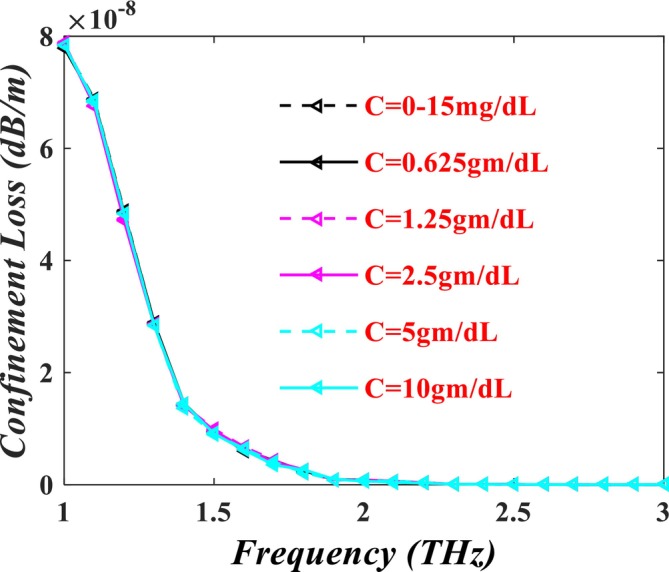
CL with THz frequency.

Figure 8 demonstrates extremely low CL within the THz frequency range, indicating strong field confinement and efficient guiding characteristics. At an operating frequency of 2.2 THz, the calculated CL remains extremely low for all investigated media with a value of 6.15 × 10^−8^ dB/m, 6.05 × 10^−8^ dB/m, 5.99 × 10^−8^ dB/m, 5.95 × 10^−8^ dB/m, and 5.92 × 10^−8^ dB/m across the considered glucose concentrations. These uniformly low loss values indicate that the proposed sensor can sustain strong optical confinement under varying analyte conditions, thereby demonstrating its suitability for THz‐based sensing applications.

Equation (6) [13] evaluates the energy distribution by performing a cross‐sectional integration over the waveguide's constituent regions (the core, cladding, and air cavities). The total modal power, which serves as the normalizing denominator, is subsequently derived by summing these individual spatial contributions.

(6) $$\eta =\frac{\int_{i}S_{z}\mathrm{dA}}{\int_{\mathrm{all}}S_{z}\mathrm{dA}}$$

**FIGURE 9:**
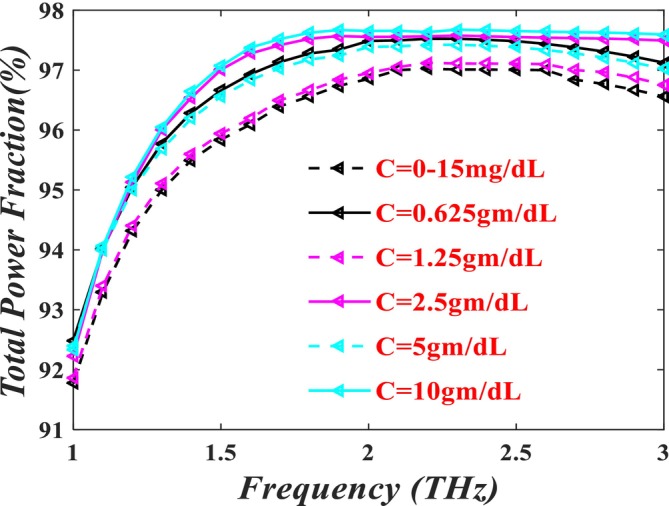
Frequency‐dependent analysis of power fraction distribution.

The power fraction (PF) for different glucose concentrations (0–15 mg·dL^−1^ to 10 g·dL^−1^) as functions of THz frequency is illustrated in Figure 9. At an operating frequency of 2.2 THz, the power fraction exhibits a gradual increase with rising glucose concentration. The PF values corresponding to glucose content are 96.56%, 97.08%, 96.85%, 97.65%, 97.74%, and 97.78%, respectively. These results indicate a consistent enhancement with increasing glucose concentration, highlighting the sensitivity of the proposed THz‐based sensing approach for glucose detection.

Ultimately, the practical sensing capability of an HPhCF is captured by its RS. This parameter physically reflects the degree to which the guided light couples with the ambient analyte. Because RS scales with power fraction defined as the optical power distributed within the sensing volume, a higher power fraction is the primary mechanism for amplifying the light‐analyte interaction and optimizing sensor response. Accordingly, the RS can be determined using the following expression (7):

(7) $$\mathrm{RS}=\frac{s_{r}}{s_{\mathrm{eff}}}\times P\%$$

In this formulation, $s_{r}$ and $s_{\mathrm{eff}}$ correspond to the sensing medium and effective modal refractive indices. The parameter P represents the fraction of total input power confined within the core. This parameter provides an indication of how effectively the guided light is concentrated within the core region. The *P* can be stated as follows (8):

(8) $$P=\frac{\int_{\text{sample}}R_{e}\left(E_{x}H_{y}-E_{y}H_{x}\right)\text{dxdy}}{\int_{\text{total}}R_{e}\left(E_{x}H_{y}-E_{y}H_{x}\right)\text{dxdy}}$$

Equation (8) evaluates the fraction of optical power confined to the core relative to the total power spanning the entire PhCF cross‐section. $E_{x}$, $E_{y}$, $H_{x}$ and $H_{y}$ denote the x‐ and y‐polarized electric and magnetic field components, respectively.

**FIGURE 10:**
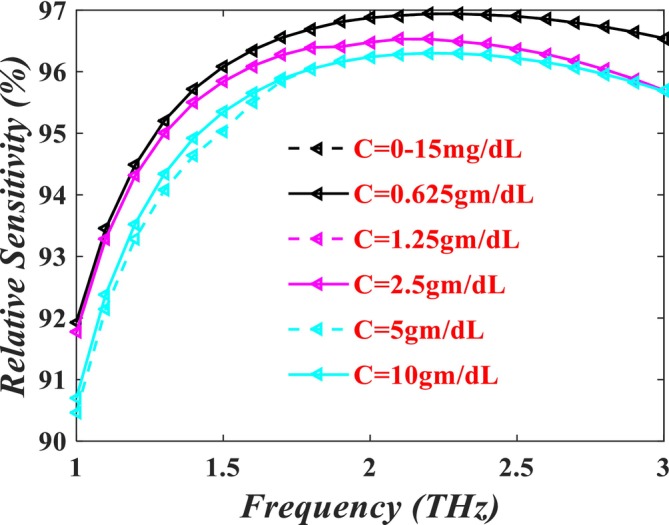
The RS demonstrates a pronounced dependence on operating frequency.

Figure 10 plots the THz RS against the analyte RI, revealing a monotonic increase in sensitivity. This behavior arises because high‐index analytes offer superior optical confinement, thereby maximizing the interaction between the propagating light and the sensing medium.

## 5 Computational Findings

The quantitative performance parameters for the selected analytes are comprehensively summarized in Table 3. Furthermore, Table 4 compares all optical performance indicators of the proposed design with previously reported sensor designs [9, 35, 36, 37, 38, 39, 40, 41, 42, 43, 44]. As evident from this comparison, the proposed structure demonstrates superior values across all optical efficiency metrics, confirming its potential as a high‐performance platform for diverse sensing applications.

**TABLE 3:** Robustness analysis: RS and CL response to ±3% geometric variations in the 2.2 THz band.

Parameters (%)	RS (%)	CL (dB/m)
	(0–15 mg·dL^−1^)	(0–15 mg·dL^−1^)
+3	97.04	6.79 × 10^−8^
Optimum	96.90	6.15 × 10^−8^
−3	95.60	5.58 × 10^−8^

**TABLE 4:** Benchmarking the sensitivity of the proposed design.

Research	Analyte	Operating frequency (THz)	EA (μm^2^)	EML	CL (dB/m)	RS (%)	Improvements (%)
[9]	Benzene	1	—	—	3.02 × 10^−8^	78.06	24.14
[36]	Benzene	1	144 000	—	2.5 × 10^−14^	63.24	53.23
[37]	RBCs	1.5	31 416	—	1.23 × 10^−13^	80.93	19.73
[35]	HCN	2	—	0.023	1.62 × 10^−9^	85.8	12.94
[38]	Benzene	1.7	—	0.028	1.23 × 10^−8^	89	8.88
[39]	NaCl	1.8	397 340	0.004	4.87 × 10^−11^	91.50	5.90
[40]	Tabun	1.8	**—**	0.009	1.71 × 10^−14^	94.4	2.65
[41]	Biochemical	2.5	97 341	0.0051	3.00 × 10^−13^	95.82	1.13
[42]	4 μmol/L bilirubin level in the blood	0.75	**—**	0.00193	2.03 × 10^−14^	98	−1.12
[43]	Albumin	4.3	**—**	0.0173	1.00 × 10^−16^	98.5	−1.62
[44]	RBCs Hemoglobin WBCs Plasma	2.7	**—**	0.015	3.80 × 10^−11^	95.8	1.15
Proposed HPhCF with hollow‐core sensor	Glucose quantity (0–15 mg·dL^−1^)	2.20	71 000	0.00632	6.15 × 10^−8^	96.90	—

To evaluate the microstructure fabrication tolerance of the sensor, we investigated the impact of a +3% deviation in geometric parameters on its optical performance, specifically focusing on RS and CL. This sensitivity analysis relies on the optimized baseline geometry (*d* = 86 μm, *d*_1_ = 365 μm), assuming a constant pitch of 455 μm across all cladding layers (*A*_1_ through *A*_5_). With the PML boundaries established at 2460 μm and 2706 μm, these simulations provide critical insight into the sensor's robustness against potential manufacturing imperfections. To evaluate the structural robustness and fabrication tolerance of the proposed HPhCF sensor, a parametric sensitivity analysis was conducted by introducing ±3% deviations to the optimized design parameters. The corresponding effects on the key optical performance metrics, namely RS and CL, are summarized in Table 3. The results demonstrate that these minor geometric variations produce only negligible changes in both RS and CL, indicating that the sensor maintains stable optical performance despite small fabrication‐induced imperfections. This low sensitivity to dimensional fluctuations confirms the reliability of the optimized design and highlights its practical manufacturability. Consequently, the proposed HPhCF sensor offers a favorable balance between fabrication feasibility and high sensing performance, making it a strong candidate for real‐world biomedical and chemical sensing applications where precision, repeatability, and operational stability are essential.

**FIGURE 11:**
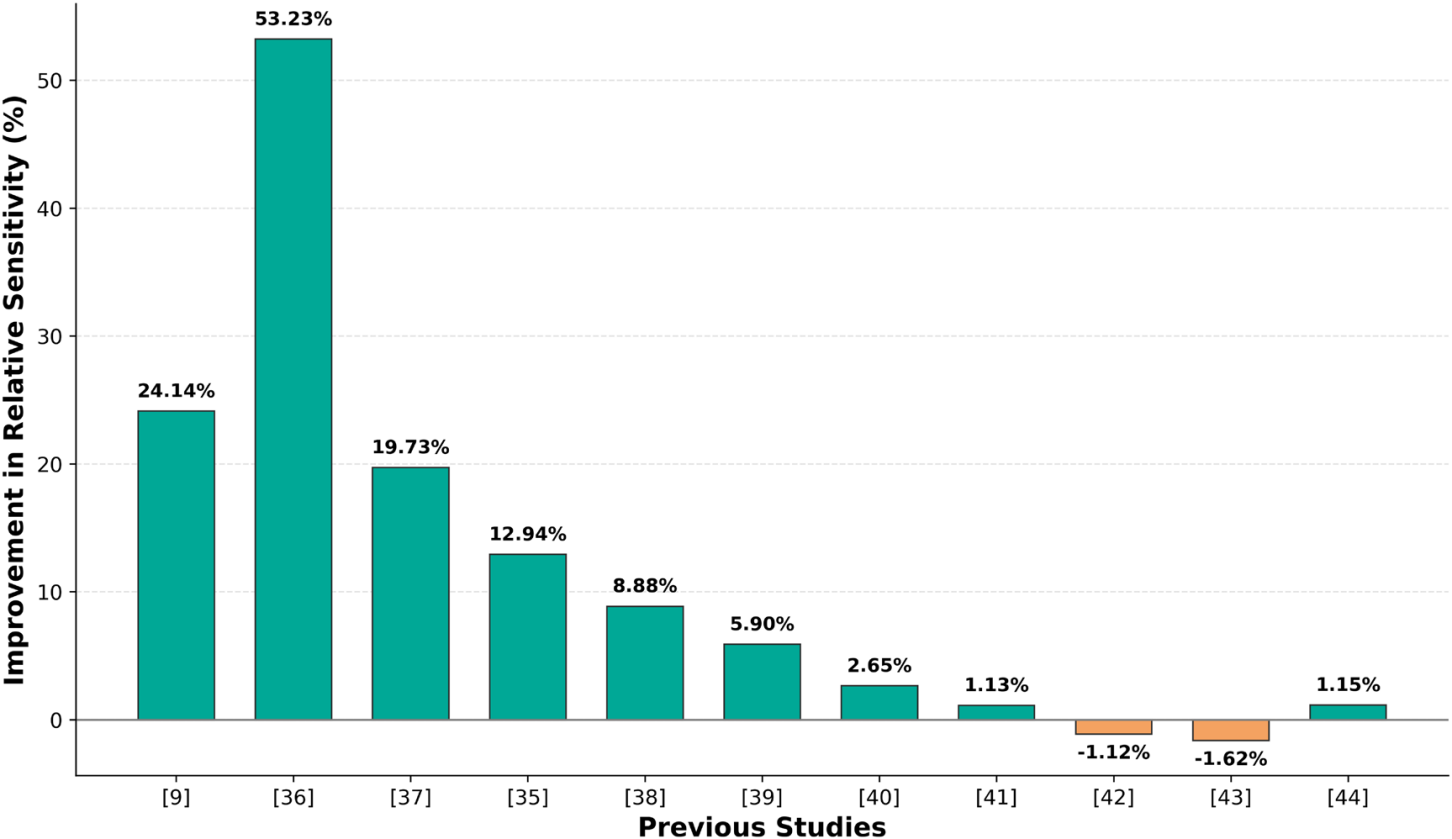
RS improvement over existing HPhCF sensors.

As summarized in Table 4, the proposed HPhCF sensor achieves a RS of 96.90%, outperforming most previously reported THz PCF sensors, with improvements ranging from 1.13% to 53.23% over the majority of existing designs. Although the sensors reported in [42, 43] exhibit slightly higher RS values (98.0% and 98.5%, respectively), the proposed sensor provides a more balanced overall performance. Specifically, it is designed for noninvasive glucose detection over a practical concentration range (0–15 mg·dL^−1^), operates at the widely adopted 2.20 THz frequency, and employs a simple hollow‐core architecture with a compact EA of 71 000 μm^2^, which facilitates fabrication while maintaining high sensitivity. Therefore, the proposed design offers an attractive trade‐off between sensing performance, structural simplicity, and practical applicability for biomedical diagnostics. Furthermore, Figure 11 graphically compares the RS of the proposed sensor with previously reported designs, clearly illustrating its competitive performance and notable improvements over the majority of existing HPhCF sensors.

From a practical manufacturing standpoint, the proposed PCF represents a structurally realistic design that aligns with current microfabrication capabilities. Recent advances in PCF fabrication, particularly sol–gel processing and related high‐precision techniques, have demonstrated the ability to produce complex air hole arrangements with excellent dimensional accuracy, making the proposed geometry technically achievable [45]. Moreover, the successful fabrication of rectangular PCF architectures at the Max Planck Institute confirms that sophisticated non‐conventional fiber geometries can be realized experimentally [46]. Previous studies have also highlighted that similar hollow‐core PCF configurations can be manufactured with only minor optimization of structural parameters, such as air hole diameter, pitch, or material composition, while preserving their optical performance [19, 38, 47]. Although experimental realization remains an important next step, the available fabrication evidence strongly indicates that the proposed structure is compatible with existing manufacturing technologies and possesses considerable potential for practical implementation in THz sensing systems.

The outstanding sensing characteristics of the proposed PCF originate from the synergistic optimization of its structural and material design [48, 49, 50]. Incorporating a hollow‐core architecture within a Zeonex background creates a pronounced refractive‐index contrast that significantly enhances the interaction between the guided THz field and the target analyte, thereby improving detection sensitivity. Simultaneously, the optimized hexagonal air hole lattice provides strong optical confinement and efficient mode guidance, resulting in an enlarged effective mode area while maintaining excellent field localization. In addition, the carefully designed cladding geometry, together with the PML, effectively suppresses electromagnetic field leakage, leading to exceptionally low CL and improved transmission efficiency over conventional PCF configurations. The combined effect of these design strategies produces a highly sensitive, low‐loss, and robust sensing platform. Consequently, the proposed PCF offers a promising solution for next‐generation biomedical sensing, particularly where high sensitivity, low propagation loss, and reliable light‐analyte interaction are critical for accurate assessment.

## 6 Algorithmic Augmentation

The convergence of machine learning (ML) with PhCF technology signifies an auspicious direction for the development of next‐generation biomedical diagnostic systems [51]. Conventional optical sensing approaches primarily rely on deterministic interpretation of spectral variations, which may limit their proficiency in capturing nonlinear dynamics embedded within biological signals. By incorporating ML‐driven computational frameworks, PhCF‐based sensing systems can significantly improve their analytical precision, sensitivity, and interpretability, thereby transforming conventional optical measurements into intelligent diagnostic tools. ML algorithms enable automated feature extraction, adaptive calibration, and intelligent signal interpretation, allowing sensing platforms to identify subtle spectral variations that may otherwise remain undetected through traditional analytical techniques [52]. In PhCF sensors, these variations may manifest as minute shifts in RI, resonance wavelength displacement, or alterations in spectral intensity patterns. ML‐based analytical frameworks are capable of identifying these patterns and establishing predictive relationships between optical responses and underlying biochemical properties. Consequently, the integration of ML with PhCF sensors facilitates the transition from passive sensing devices to self‐learning analytical systems capable of continuous optimization and autonomous decision‐making.

Within the context of early diabetes detection, ML‐assisted PhCF sensing platforms offer significant potential for identifying extremely small biochemical changes in biological fluids, such as blood, urine, or interstitial fluid. These changes may include fluctuations in glucose concentration, RI variations, or the presence of glucose‐related biomarkers, which are critical indicators of metabolic disorders [53]. The spectral data can be effectively processed using various machine learning classifiers, such as convolutional neural networks, support vector machines, and random forests generated by PhCF sensors, and classify physiological states with high accuracy [54, 55]. By learning from previously observed spectral signatures, these algorithms can differentiate between normal physiological conditions and diabetic profiles in near real time, thereby enabling rapid, noninvasive, and cost‐efficient disease screening mechanisms.

Beyond conventional ML models, the integration of advanced learning paradigms further enhances the capability of photonic biosensing platforms [56]. Modern intelligent frameworks such as Model‐Agnostic Meta‐Learning (MAML++), Temporal Fusion Transformer (TFT), Meta‐Ensemble learning strategies, Bayesian Hyperparameter Optimization (HPO), and Real‐Time Data Augmentation (RTDA) provide powerful mechanisms for improving predictive performance, model adaptability, and robustness in biomedical sensing environments [57, 58, 59, 60, 61]. The MAML++ meta‐learning framework enables fast adaptation of learning models to personalize spectral characteristics generated by PhCF sensors. This facility holds significant value in biomedical applications where datasets are comparatively restricted and patient variability is high. Through meta‐learning, models can acquire generalized knowledge during training and subsequently adapt to new sensing conditions using only a small number of samples [62]. Such adaptability significantly mitigates the challenges associated with data scarcity and inter‐patient variability.

Similarly, the TFT architecture introduces advanced attention mechanisms capable of modeling temporal dependencies in sequential biomedical signals. When applied to spectral outputs generated by PhCF sensors, TFT can capture time‐dependent patterns in glucose‐related spectral variations, enabling accurate trend forecasting and disease progression monitoring. The interpretability features embedded within TFT also allow researchers and clinicians to understand which spectral components contribute most significantly to diagnostic predictions. Another powerful strategy is the Meta‐Ensemble learning framework, which combines multiple predictive models to improve overall system performance [62]. By integrating the outputs of diverse learning algorithms, Meta‐Ensemble techniques reduce prediction variance and enhance generalization capability across heterogeneous biomedical datasets [63]. This approach ensures stable, reliable diagnostic performance even when sensing conditions vary across biological samples or measurement environments [64].

Complementing these strategies, Bayesian HPO introduces a probabilistic optimization mechanism that automatically identifies optimal model configurations. This approach systematically explores the parameter space of ML models while accounting for uncertainty, thereby improving predictive performance under dynamic sensing conditions. Simultaneously, RTDA expands the diversity of training datasets by generating synthetic spectral samples that preserve physiological realism. Such augmentation strategies are particularly beneficial in biomedical sensing scenarios where acquiring large quantities of labeled data may be challenging. When integrated within a PhCF‐based sensing platform, these ML‐driven analytical layers collectively establish an intelligent diagnostic ecosystem capable of detecting extremely subtle RI variations induced by changes in glucose concentration. The combination of photonic sensing and adaptive computational intelligence significantly enhances the sensitivity, reliability, and real‐time analytical capability of the system [65, 66, 67, 68, 69, 70].

**FIGURE 12:**
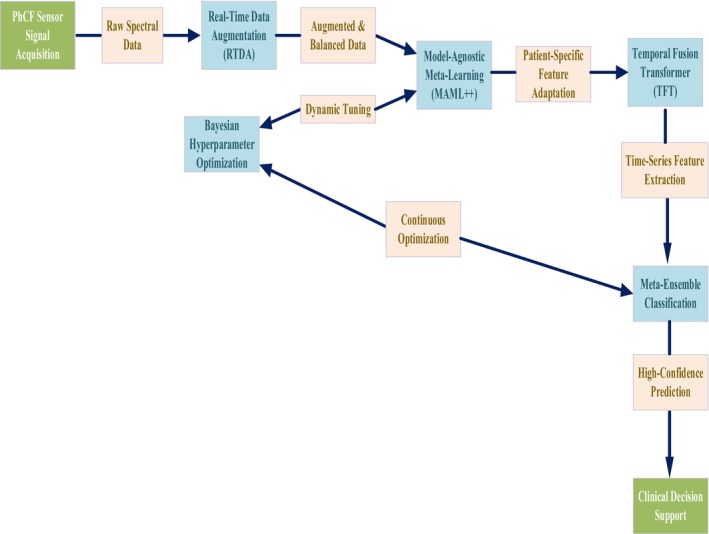
Technical flowchart of the ML‐Integrated PhCF diagnostic framework, where the green block presents input/output interfaces, the blue block presents computational processes, and the orange block presents data flow & interaction.

Ultimately, the synergy between photonic hardware and self‐optimizing machine learning architecture paves the way for the development of advanced healthcare technologies. Such systems hold immense potential for enabling real‐time, noninvasive diabetes monitoring, early‐stage disease detection, and predictive medical interventions. By bridging the gap between optical biosensing and intelligent data‐driven analytics, the synthesis of ML with PhCF technology reflects a considerable advancement toward personalized healthcare and next‐generation biomedical diagnostics. A combined figure is presented in Figure 12, where the workflow begins with raw spectral acquisition from the PhCF sensor. Data undergoes RTDA to address data imbalance. The processed data enters the core analytical engine, where MAML++ facilitates rapid adaptation to individual patient profiles, and the TFT extracts time‐dependent features. A Meta‐Ensemble approach synthesizes these inputs for final classification, while Bayesian HPO operates continuously in the background to ensure model parameters are optimized for the current biosensing environment.

## 7 Limitations and Future Work

Although the proposed THz‐based PCF sensor demonstrates excellent numerical performance, several limitations should be acknowledged. First, the clinical translation of THz‐PCF sensors for early‐stage diabetes diagnosis using urinary glucose remains challenging because of current technological constraints, fabrication complexity, and the limited availability of clinically validated THz sensing platforms. Therefore, the present study is intended to establish a rigorous theoretical and computational foundation rather than introduce a clinically deployable diagnostic device. The FEM simulations demonstrate that the proposed sensor possesses high RS together with low CL and EML, providing strong evidence of its potential for future biomedical sensing applications. Second, although this work presents a comprehensive framework for integrating advanced ML techniques, including MAML++, TFT, Meta‐Ensemble learning, Bayesian HPO, and RTDA, the ML component is intentionally conceptual and has not been experimentally validated in the current study. At present, no publicly available benchmark dataset exists containing THz spectral responses of urinary glucose with sufficient sample diversity to enable reliable feature extraction, model training, validation, and comparative evaluation of these advanced learning algorithms. Consequently, implementing and quantitatively validating the proposed ML framework would be scientifically premature and beyond the scope of this work. Instead, the proposed ML architecture is presented as a forward‐looking computational framework illustrating how intelligent data analytics can be integrated with future THz‐PCF sensing systems once experimentally acquired spectral datasets become available. As a future direction, we plan to fabricate and experimentally characterize the proposed sensor through collaboration with biomedical and photonics researchers, generating clinically relevant THz spectral datasets under controlled laboratory conditions. These datasets will enable systematic feature engineering, standardized training and validation protocols, and comprehensive benchmarking of the proposed ML models. In addition, future investigations will explore biocompatible low‐loss fiber materials, optimized sensor geometries, fabrication tolerance analysis, and clinical validation using real biological samples, thereby bridging the gap between numerical design, intelligent data analytics, and practical biomedical implementation. Collectively, this staged research strategy establishes a scientifically rigorous pathway toward the development of intelligent, reliable, and clinically applicable THz‐enabled biomedical diagnostic systems.

## 8 Conclusion

We present a novel HPhCF designed for THz biochemical sensing. Characterized over a 1–3 THz bandwidth, the waveguide successfully quantifies varying glucose concentrations in urine, demonstrating strong potential for clinical diagnostic applications. Structurally, the HPhCF incorporates a simple rectangular array configuration, which facilitates practical and cost‐effective fabrication. In addition, the design exhibits negligible EML and CL, which contributes to improved signal stability and measurement accuracy. With its exceptional sensitivity and low loss, the HPhCF stands out as a practical tool for the rapid, noninvasive screening of urinary glucose in early‐stage diabetes. Crucially, the platform is not limited to biomedical diagnostics; its design is equally applicable to diverse chemical and biochemical sensing paradigms.

## Author Contributions

**Mohammad Abdullah‐Al‐Shafi:** conceptualization, data curation, formal analysis, investigation, writing – original draft. **Shuvo Sen:** methodology, resources, software analysis, validation, visualization. **Mashiyat Mubassera:** visualization, resources, methodology, investigation. **Md. Tanvir Hossain Hawlader:** methodology, data curation, resources, visualization.

## Funding

The authors have nothing to report.

## Ethics Statement

The authors have nothing to report.

## Consent

The authors provide consent for publication. The authors agreed to submit the paper to this journal.

## Conflicts of Interest

The authors declare no conflicts of interest.

## Data Availability

The data supporting the findings of this study are available from the corresponding author upon reasonable request.

## References

[1] E. Standl , K. Khunti , T. B. Hansen , and O. Schnell , “The Global Epidemics of Diabetes in the 21st Century: Current Situation and Perspectives,” European Journal of Preventive Cardiology 26, no. 2_suppl (2019): 7–14. 10.1177/2047487319881021 31766915

[2] H. Teymourian , A. Barfidokht , and J. Wang , “Electrochemical Glucose Sensors in Diabetes Management: An Updated Review (2010–2020),” Chemical Society Reviews 49, no. 21 (2020): 7671–7709. 33020790 10.1039/d0cs00304b

[3] M. Abdullah‐Al‐Shafi , “COVID‐19 Pandemic: A Viewpoint From Asia,” Bulletin of the National Research Centre 44, no. 1 (2020): 80. 32501384 10.1186/s42269-020-00337-5 PMC7256341

[4] A. S. Bolla and R. Priefer , “Blood Glucose Monitoring‐An Overview of Current and Future Noninvasive Devices,” Diabetes and Metabolic Syndrome: Clinical Research and Reviews 14, no. 5 (2020): 739–751. 10.1016/j.dsx.2020.05.016 32497964

[5] S. A. Pullano , M. Greco , M. G. Bianco , D. Foti , A. Brunetti , and A. S. Fiorillo , “Glucose Biosensors in Clinical Practice: Principles, Limits and Perspectives of Currently Used Devices,” Theranostics 12, no. 2 (2022): 493–511. 34976197 10.7150/thno.64035 PMC8692922

[6] P. Jain , A. M. Joshi , S. P. Mohanty , and L. R. Cenkeramaddi , “Noninvasive Glucose Measurement Technologies: Recent Advancements and Future Challenges,” IEEE Access 12 (2024): 61907–61936.

[7] M. Abdullah‐Al‐Shafi , S. Sen , and M. Mubassera , “Insecure Food Additive Sensing With Photonic Crystal Fiber in Terahertz Regime,” Journal of Modern Optics 71, no. 19–21 (2024): 762–770.

[8] M. Abdullah‐Al‐Shafi and S. Sen , “A Sophisticated Terahertz Photonic Crystal Fiber Sensor Design for Highly Accurate Detection of Kerosene Mixtures,” Advanced Physics Research 4 (2025): 2500025.

[9] M. Abdullah‐Al‐Shafi and S. Sen , “Design and Analysis of a Chemical Sensing Octagonal Photonic Crystal Fiber (O‐PCF) Based Optical Sensor With High Relative Sensitivity for Terahertz (THz) Regime,” Sensing and Bio‐Sensing Research 29 (2020): 100372.

[10] S. Sen and M. Abdullah‐Al‐Shafi , “Emphasis on Sensitivity and Accuracy: Design and Optimization of a High‐Sensitivity Terahertz Photonic Crystal Fiber Sensor for Precision Analysis of Petrochemical‐Based Adulterants in Hydrocarbon Mixtures,” Sensing and Bio‐Sensing Research 49 (2025): 100823.

[11] M. S. Hossain , N. Hussain , Z. Hossain , et al., “Performance Analysis of Alcohols Sensing With Optical Sensor Procedure Using Circular Photonic Crystal Fiber (C‐PCF) in the Terahertz Regime,” Sensing and Bio‐Sensing Research 35 (2022): 100469.

[12] S. Sen , M. Abdullah‐Al‐Shafi , and M. A. Kabir , “Hexagonal Photonic Crystal Fiber (H‐PCF) Based Optical Sensor With High Relative Sensitivity and Low Confinement Loss for Terahertz (THz) Regime,” Sensing and Bio‐Sensing Research 30 (2020): 100377.

[13] S. Sen , M. Abdullah‐Al‐Shafi , A. S. Sikder , M. S. Hossain , and M. M. Azad , “Zeonex Based Decagonal Photonic Crystal Fiber (D‐PCF) in the Terahertz (THz) Band for Chemical Sensing Applications,” Sensing and Bio‐Sensing Research 31 (2021): 100393.

[14] M. Abdullah‐Al‐Shafi and S. Sen , “Design of a Low Material Loss and Larger Effective Area Based Photonic Crystal Fiber for Communication Applications in Terahertz (THz) Waveguide,” Sensing and Bio‐Sensing Research 31 (2021): 100400.

[15] M. S. H. Mollah , M. Abdullah‐Al‐Shafi , M. S. Hossain , and S. Sen , “An Ultra‐Low Material Loss Ellipse Core‐Based Photonic Crystal Fiber for Terahertz Wave Guiding: Design and Analysis,” Journal of Computational Electronics 20, no. 4 (2021): 1541–1548.

[16] M. Abdullah‐Al‐Shafi , N. Akter , S. Sen , and M. S. Hossain , “Design and Performance Analysis of Background Material of Zeonex Based High Core Power Fraction and Extremely Low Effective Material Loss of Photonic Crystal Fiber in the Terahertz (THz) Wave Pulse for Many Types of Communication Areas,” Optik 243 (2021): 167519.

[17] S. Sawraj , D. Kumar , R. Pravesh , et al., “PCF‐Based Sensors for Biomedical Applications: A Review,” IEEE Transactions on Nanobioscience 24, no. 2 (2024): 157–164. 10.1109/TNB.2024.3462748 39288060

[18] D. N. Alhamss , S. A. Taya , A. H. Almawgani , et al., “Numerical Analysis of a Photonic Crystal Fiber‐Based Biosensor for the Detection of Vibrio Cholera and *Escherichia coli* Bacteria in the THz Regime,” Physica Status Solidi A 221, no. 2 (2024): 2300622.

[19] M. S. Islam , J. Sultana , A. A. Rifat , A. Dinovitser , B. W. H. Ng , and D. Abbott , “Terahertz Sensing in a Hollow Core Photonic Crystal Fiber,” IEEE Sensors Journal 18, no. 10 (2018): 4073–4080.

[20] W. Jiang , Q. Zhou , J. He , et al., “Terahertz Communications and Sensing for 6G and Beyond: A Comprehensive Review,” IEEE Communications Surveys & Tutorials 26, no. 4 (2024): 2326–2381.

[21] X. Huang , Y. Wu , Y. Ni , H. Xu , and Y. He , “Global, Regional, and National Burden of Type 2 Diabetes Mellitus Caused by High BMI From 1990 to 2021, and Forecasts to 2045: Analysis From the Global Burden of Disease Study 2021,” Frontiers in Public Health 13 (2025): 1515797. 39916706 10.3389/fpubh.2025.1515797 PMC11798972

[22] Y. Li , Y. Liu , S. Liu , et al., “Diabetic Vascular Diseases: Molecular Mechanisms and Therapeutic Strategies,” Signal Transduction and Targeted Therapy 8, no. 1 (2023): 152. 37037849 10.1038/s41392-023-01400-z PMC10086073

[23] O. Hoffstad , N. Mitra , J. Walsh , and D. J. Margolis , “Diabetes, Lower‐Extremity Amputation, and Death,” Diabetes Care 38, no. 10 (2015): 1852–1857. 26203063 10.2337/dc15-0536

[24] R. Pandey , S. K. Paidi , T. A. Valdez , et al., “Noninvasive Monitoring of Blood Glucose With Raman Spectroscopy,” Accounts of Chemical Research 50, no. 2 (2017): 264–272. 28071894 10.1021/acs.accounts.6b00472 PMC5896772

[25] A. Hina and W. Saadeh , “Noninvasive Blood Glucose Monitoring Systems Using Near‐Infrared Technology—A Review,” Sensors (Basel) 22, no. 13 (2022): 4855. 35808352 10.3390/s22134855 PMC9268854

[26] L. Tang , S. J. Chang , C. J. Chen , and J. T. Liu , “Noninvasive Blood Glucose Monitoring Technology: A Review,” Sensors (Basel) 20, no. 23 (2020): 6925. 33291519 10.3390/s20236925 PMC7731259

[27] N. B. Davison , C. J. Gaffney , J. G. Kerns , and Q. D. Zhuang , “Recent Progress and Perspectives on Noninvasive Glucose Sensors,” Diabetology 3, no. 1 (2022): 56–71.

[28] N. C. Godja and F. D. Munteanu , “Hybrid Nanomaterials: A Brief Overview of Versatile Solutions for Sensor Technology in Healthcare and Environmental Applications,” Biosensors 14, no. 2 (2024): 67. 38391986 10.3390/bios14020067 PMC10887000

[29] G. Woyessa , J. K. Pedersen , A. Fasano , et al., “Zeonex‐PMMA Microstructured Polymer Optical FBGs for Simultaneous Humidity and Temperature Sensing,” Optics Letters 42, no. 6 (2017): 1161–1164. 28295073 10.1364/OL.42.001161

[30] A. H. M. Almawgani , S. A. Taya , M. G. Daher , I. Colak , F. Wu , and S. K. Patel , “Detection of Glucose Concentration Using a Surface Plasmon Resonance Biosensor Based on Barium Titanate Layers and Molybdenum Disulphide Sheets,” Physica Scripta 97, no. 6 (2022): 065501, 10.1088/1402-4896/ac68ad.

[31] Y. Shen , Z. Wang , Z. Wang , et al., “Thermally Drawn Multifunctional Fibers: Toward the Next Generation of Information Technology,” InfoMat 4, no. 7 (2022): e12318.

[32] H. Wang , W. Zhang , D. Ladika , et al., “Two‐Photon Polymerization Lithography for Optics and Photonics: Fundamentals, Materials, Technologies, and Applications,” Advanced Functional Materials 33, no. 39 (2023): 2214211.

[33] D. N. Alhamss , A. H. Almawgani , A. R. Alhawari , et al., “A Terahertz Photonic Crystal Fiber Sensor for Enhanced Protein Level Detection,” Plasmonics 20, no. 8 (2025): 6049–6059.

[34] S. Yadav , S. Singh , P. Lohia , A. Umar , and D. K. Dwivedi , “Delineation of Profoundly Birefringent Nonlinear Photonic Crystal Fiber in Terahertz Frequency Regime,” Journal of Optical Communications 46, no. 1 (2025): 41–49.

[35] M. S. Islam , J. Sultana , A. Dinovitser , K. Ahmed , B. W. H. Ng , and D. Abbott , “Sensing of Toxic Chemicals Using Polarized Photonic Crystal Fiber in the Terahertz Regime,” Optics Communications 426 (2018): 341–347.

[36] M. M. Hasan , S. Sen , M. J. Rana , et al., “Heptagonal Photonic Crystal Fiber Based Chemical Sensor in THz Regime,” (2019), In 2019 Joint 8th International Conference on Informatics, Electronics & Vision (ICIEV) and 2019 3rd International Conference on Imaging, Vision & Pattern Recognition (icIVPR), (40–44). IEEE.

[37] K. Ahmed , F. Ahmed , S. Roy , et al., “Refractive Index‐Based Blood Components Sensing in Terahertz Spectrum,” IEEE Sensors Journal 19, no. 9 (2019): 3368–3375.

[38] M. A. Habib , M. S. Anower , L. F. Abdulrazak , and M. S. Reza , “Hollow Core Photonic Crystal Fiber for Chemical Identification in Terahertz Regime,” Optical Fiber Technology 52 (2019): 101933.

[39] A. A. Bulbul , M. B. Hossain , R. Dutta , and M. Hassan , “Zeonex‐Based Tetra‐Rectangular Core‐Photonic Crystal Fiber for NaCl Detection,” Nanoscience and Nanotechnology ‐ Asia 11, no. 4 (2021): 112–120.

[40] M. B. Hossain , E. Podder , A. A. M. Bulbul , and H. S. Mondal , “Bane Chemicals Detection Through Photonic Crystal Fiber in THz Regime,” Optical Fiber Technology 54 (2020): 102102.

[41] A. A. M. Bulbul , A. Z. Kouzani , M. P. Mahmud , and A. A. Nahid , “Design and Numerical Analysis of a Novel Rectangular PCF (R‐PCF)‐Based Biochemical Sensor (BCS) in the THz Regime,” International Journal of Optics 2021, no. 1 (2021): 5527724.

[42] A. R. Elhelw , M. S. S. Ibrahim , A. N. Z. Rashed , A. E. N. A. Mohamed , M. F. O. Hameed , and S. S. Obayya , “Highly Sensitive Bilirubin Biosensor Based on Photonic Crystal Fiber in Terahertz Region,” Photonics 10, no. 1 (2023): 68.

[43] M. M. Rahman , F. A. Mou , M. I. H. Bhuiyan , and M. R. Islam , “Refractometric Sensing of Protein in Urine by the Photonic Crystal Fiber Biosensor in THz Regime,” International Journal of Optics 2023, no. 1 (2023): 6652333.

[44] M. M. Eid , M. A. Habib , M. S. Anower , and A. N. Z. Rashed , “Hollow Core Photonic Crystal Fiber (PCF)–Based Optical Sensor for Blood Component Detection in Terahertz Spectrum,” Brazilian Journal of Physics 51, no. 4 (2021): 1017–1025.

[45] M. F. Ferreira , M. Rehan , V. Mishra , et al., “Roadmap on Specialty Optical Fibers,” Journal of Physics: Photonics 7, no. 1 (2025): 012501.

[46] P. S. J. Russell , R. Beravat , and G. K. L. Wong , “Helically Twisted Photonic Crystal Fibres,” Philosophical Transactions of the Royal Society A: Mathematical, Physical and Engineering Sciences 375, no. 2087 (2017): 20150440. 10.1098/rsta.2015.0440 PMC5247484 28069771

[47] N. A. Mohammed , O. E. Khedr , E. S. M. El‐Rabaie , and A. A. Khalaf , “Early Detection of Brain Cancers Biomedical Sensor With Low Losses and High Sensitivity in the Terahertz Regime Based on Photonic Crystal Fiber Technology,” Optical and Quantum Electronics 55, no. 3 (2023): 230.

[48] Z. X. Peng , B. X. Li , and C. S. Deng , “Ultrahigh‐Q Fano Resonance in a Cavity‐Waveguide Coupled System Based on Second‐Order Topological Photonic Crystals With Elliptical Holes,” Optics and Laser Technology 181 (2025): 111617.

[49] T. Li , J. Bu , Y. Yang , and S. Zhong , “A Smartphone‐Assisted One‐Step Bicolor Colorimetric Detection of Glucose in Neutral Environment Based on Molecularly Imprinted Polymer Nanozymes,” Talanta 267 (2024): 125256. 37801931 10.1016/j.talanta.2023.125256

[50] Y. Yan , Y. F. Jiang , B. X. Li , and C. S. Deng , “Controlling Dual Fano Resonance Lineshapes Based on an Indirectly Coupled Double‐Nanobeam‐Cavity Photonic Molecule,” Journal of Lightwave Technology 42, no. 2 (2023): 732–739.

[51] C. Wang , T. He , H. Zhou , Z. Zhang , and C. Lee , “Artificial Intelligence Enhanced Sensors‐Enabling Technologies to Next‐Generation Healthcare and Biomedical Platform,” Bioelectronic Medicine 9, no. 1 (2023): 17. 37528436 10.1186/s42234-023-00118-1 PMC10394931

[52] S. Kaziz , F. Echouchene , and M. H. Gazzah , “Optimizing PCF‐SPR Sensor Design Through Taguchi Approach, Machine Learning, and Genetic Algorithms,” Scientific Reports 14, no. 1 (2024): 7837. 38570590 10.1038/s41598-024-55817-9 PMC10991260

[53] C. Moonla , M. Reynoso , A. Y. Chang , T. Saha , S. Surace , and J. Wang , “Microneedle‐Based Multiplexed Monitoring of Diabetes Biomarkers: Capabilities Beyond Glucose Toward Closed‐Loop Theranostic Systems,” ACS Sensors 10 (2025): 5363–5379. 40460286 10.1021/acssensors.5c00652 PMC12379172

[54] R. Kamalraj , S. Neelakandan , M. R. Kumar , V. C. S. Rao , R. Anand , and H. Singh , “Interpretable Filter Based Convolutional Neural Network (IF‐CNN) for Glucose Prediction and Classification Using PD‐SS Algorithm,” Measurement 183 (2021): 109804.

[55] S. Lekha and M. Suchetha , “Real‐Time Noninvasive Detection and Classification of Diabetes Using Modified Convolution Neural Network,” IEEE Journal of Biomedical and Health Informatics 22, no. 5 (2017): 1630–1636. 28961131 10.1109/JBHI.2017.2757510

[56] M. A. Amirabadi , S. A. Nezamalhosseini , M. H. Kahaei , and L. R. Chen , “A Comprehensive Survey on Machine and Deep Learning for Optical Communications,” (2025), IEEE Access.

[57] K. Singh and D. Malhotra , “IRAM–NET Model: Image Residual Agnostics Meta‐Learning‐Based Network for Rare de Novo Glioblastoma Diagnosis,” Neural Computing and Applications 36, no. 34 (2024): 21465–21485.

[58] S. Madan , M. Lentzen , J. Brandt , D. Rueckert , M. Hofmann‐Apitius , and H. Fröhlich , “Transformer Models in Biomedicine,” BMC Medical Informatics and Decision Making 24, no. 1 (2024): 214. 39075407 10.1186/s12911-024-02600-5 PMC11287876

[59] Y. Guo , C. Chang , C. Chen , et al., “Meta‐Ensemble Learning With Adaptive Sampling for Imbalanced Medical Raman Spectroscopy Data,” Applied Soft Computing 176 (2025): 113142.

[60] A. A. Abdullah , M. M. Hassan , and Y. T. Mustafa , “A Review on Bayesian Deep Learning in Healthcare: Applications and Challenges,” IEEE Access 10 (2022): 36538–36562.

[61] C. Halmich , L. Höschler , C. Schranz , and C. Borgelt , “Data Augmentation of Time‐Series Data in Human Movement Biomechanics: A Scoping Review,” PLoS One 20, no. 7 (2025): e0327038. 40591732 10.1371/journal.pone.0327038 PMC12212866

[62] M. Abdullah‐Al‐Shafi , G. Sorwar , A. R. Alaei , and M. Ahmed , “GHDFloodNet: An Advanced Model for Improved Short‐Term Flood Forecasting,” Water 18, no. 13 (2026): 1580.

[63] T. A. Shaikh , T. Rasool , P. Verma , and W. A. Mir , “A Fundamental Overview of Ensemble Deep Learning Models and Applications: Systematic Literature and State of the Art,” Annals of Operations Research (2024): 1–77.37361099

[64] M. Abdullah‐Al‐Shafi and S. Sen , “A Next‐Generation Optimized Hexagonal Photonic Crystal Fiber Biosensor for High‐Precision Early Brain Tumor Detection,” Biomedical Materials & Devices (2026): 1–19.

[65] M. A. Butt , “Surface Plasmon Resonance‐Based Biodetection Systems: Principles, Progress and Applications—A Comprehensive Review,” Biosensors 15, no. 1 (2025): 35. 39852086 10.3390/bios15010035 PMC11763797

[66] M. A. Butt , X. Mateos , and R. Piramidowicz , “Photonics Sensors: A Perspective on Current Advancements, Emerging Challenges, and Potential Solutions,” Physics Letters A 516 (2024): 129633.

[67] S. Sen , M. Abdullah‐Al‐Shafi , M. Mubassera , and S. A. Chowdhury , “Terahertz Photonic Crystal Fiber Architecture for Ultra‐Sensitive Detection of Brain Tumour Cells,” Chemical Physics Impact 12 (2026): 101049.

[68] M. Abdullah‐Al‐Shafi , S. Sen , and M. Mubassera , “Machine Learning Assisted Malaria Detection Using Photonic Crystal Fiber Optical Sensors,” Scientific Reports 16 (2026): 8320. 41673077 10.1038/s41598-026-37709-2 PMC12966299

[69] S. Sen , M. Abdullah‐Al‐Shafi , M. Mubassera , and M. T. H. Hawlader , “A High‐Sensitivity Photonic Crystal Fiber Biosensor for Malaria Detection,” Sensing and Bio‐Sensing Research 51 (2026): 100963.

[70] D. Leykam , H. Xue , B. Zhang , and Y. D. Chong , “Limitations and Possibilities of Topological Photonics,” Nature Reviews Physics 8, no. 1 (2026): 55–64.

